# 3-[(*E*)-(2,4-Dichloro­pbenzyl­idene)amino]­benzoic acid

**DOI:** 10.1107/S1600536810051470

**Published:** 2010-12-18

**Authors:** Muhammad Akmal, Waseeq Ahmad Siddiqui, M. Nawaz Tahir, Adnan Ashraf, Farhat Nosheen

**Affiliations:** a Department of Chemistry, University of Sargodha, Sargodha, Pakistan; b Department of Physics, University of Sargodha, Sargodha, Pakistan

## Abstract

In the crystal of the title compound, C_14_H_9_Cl_2_NO_2_, inversion-related dimers with *R*_2_^2^(8) ring motifs are formed by inter­molecular O—H⋯O hydrogen bonding. The 3-amino­benzoic acid group and the 2,4-dichlobenzaldehyde moiety subtend a dihedral angle of 55.10 (2)°. The H atom of the carboxyl group is disordered over two sites with equal occupancies.

## Related literature

For our project on the synthesis of various Schiff bases of 2,4-dichloro­benzaldehyde, see: Hayat *et al.* (2010 ▶). For graph-set notation, see: Bernstein *et al.* (1995 ▶).
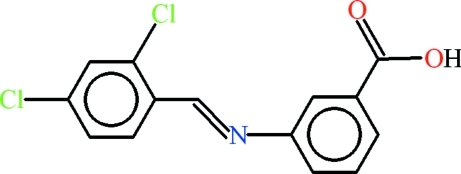


## Experimental

### 

#### Crystal data

C_14_H_9_Cl_2_NO_2_
*M* *_r_* = 294.12
Triclinic, 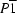

*a* = 7.4065 (2) Å
*b* = 7.6176 (3) Å
*c* = 11.5330 (4) Å
α = 86.946 (2)°
β = 80.433 (1)°
γ = 85.833 (2)°
*V* = 639.38 (4) Å^3^
*Z* = 2
Mo *K*α radiation
μ = 0.50 mm^−1^
*T* = 296 K
0.32 × 0.24 × 0.20 mm

#### Data collection

Bruker Kappa APEXII CCD diffractometer
Absorption correction: multi-scan (*SADABS*; Bruker, 2005 ▶) *T*_min_ = 0.903, *T*_max_ = 0.932
9596 measured reflections
2293 independent reflections
2048 reflections with *I* > 2σ(*I*)
*R*_int_ = 0.023

#### Refinement

*R*[*F* ^2^ > 2σ(*F* ^2^)] = 0.031
*wR*(*F* ^2^) = 0.084
*S* = 1.05
2293 reflections
175 parameters
H-atom parameters constrained
Δρ_max_ = 0.22 e Å^−3^
Δρ_min_ = −0.31 e Å^−3^

### 

Data collection: *APEX2* (Bruker, 2009 ▶); cell refinement: *SAINT* (Bruker, 2009 ▶); data reduction: *SAINT*; program(s) used to solve structure: *SHELXS97* (Sheldrick, 2008 ▶); program(s) used to refine structure: *SHELXL97* (Sheldrick, 2008 ▶); molecular graphics: *ORTEP-3 for Windows* (Farrugia, 1997 ▶) and *PLATON* (Spek, 2009 ▶); software used to prepare material for publication: *WinGX* (Farrugia, 1999 ▶) and *PLATON*.

## Supplementary Material

Crystal structure: contains datablocks global, I. DOI: 10.1107/S1600536810051470/bg2380sup1.cif

Structure factors: contains datablocks I. DOI: 10.1107/S1600536810051470/bg2380Isup2.hkl

Additional supplementary materials:  crystallographic information; 3D view; checkCIF report

## Figures and Tables

**Table 1:** Hydrogen-bond geometry (Å, °)

*D*—H⋯*A*	*D*—H	H⋯*A*	*D*⋯*A*	*D*—H⋯*A*
O1—H1⋯O2^i^	0.82	1.83	2.6364 (17)	170
O2—H2⋯O1^i^	0.82	1.84	2.6364 (17)	162

Symmetry code: (i) 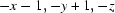.

**Fig. 1.**
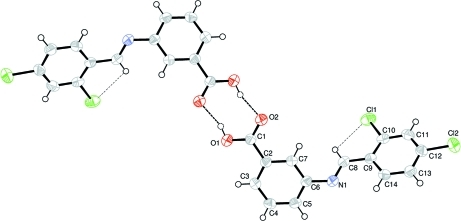
View of the H-bonded dimeric unit, with the atom numbering scheme. Only one of the two disordered carboxylate hydrogens is shown (the one attached to O1, in broken circles; the one attached to O2, omited for clarity). Thermal ellipsoids are drawn at the 50% probability level. Thin dotted lines represent intramolecular H-bonds, while intermoleculer ones, linking dimers through an R22(8) ring motif are shown as thick broken lines.

## supplementary crystallographic information

### Comment

The title compound (I, Fig. 1) is being reported as a part of our project related to the synthesis of various Schiff bases of 2,4-dichlorobenzaldehyde (Hayat *et al.*, 2010) and then their metal complexation.

In the title compound, C_14_H_9_Cl_2_NO_2_, the 3-aminobenzoic group A (C1—C7/N1/O1/O2, ring centroid Cg1) and 2,4-dichlobenzaldehyde moiety B (C7—C14/CL1/CL2, ring centroid Cg2) are planar with r. m. s. deviation of 0.0200 and 0.0352 Å, respectively. The A/B dihedral angle is 55.10 (2)°. An S(5) ring motif is formed due to an intramolecular H-bond of the C—H···Cl type (Fig. 1 and Table 1). The title compound consists of H-bonded dimers due to intermolecular H-bondings of the O—H···O type (Table 1, Fig. 1) with *R*_2_^2^(8) ring motifs (Bernstein *et al.*, 1995). There exist π–π interactions between phenyl rings, connecting dimers into a 3D arrangement. The ring in the aminobenzoic group (Cg1) interacts with its symmetrry related ones Cg1^i^ and Cg1^ii^, (i: -*x*, -*y*, - *z*, ii: -*x*, 1 - *y*, -*z*, intercentroid distances 4.1122 (9), 4.4517 (9)Å; interplanar separations: 3.3935 (6), 3.4518 (6)Å, respectively); the one in the dichlobenzaldehyde group (Cg2), in turn, interacts with Cg2^iii^ and Cg2^iv^, (iii: -*x*, -*y*, 1 - *z*, iv: 1- *x*, -*y*, 1 - *z*; intercentroid distances 4.2926 (9), 4.0256 (9) Å; interplanar separations: 3.5346 (6), 3.5224 (6) Å, respectively). The H-atom of the carboxylate group is disordered over two sites with equal occupancy ratio.

### Experimental

A mixture of *m*-aminobenzoic acid (0.25 g, 1.82 mmol) and 2,4-dichlorobenzaldehyde (0.32 g, 1.82 mmol) in absolute ethanol (20 ml) with few drops of acetic acid was heated to reflux (2 h), cooled to room temperature and filtered. The yellow precipitates were washed with the same solvent and dried at room temperature to get 0.47 g of the title compound (1.62 mmol, 89%). The crude material was dissolved in methanol and subjected to slow evaporation. Light yellow prisms of (I) were obtained after 48 h.

### Refinement

The H-atom of carboxylate is disordered over two sites with equal occupancy ratio. Initially the coordinates and multiplicity of both H-atoms were refined, which resulted with equal occupancy ratio.

The C–H atoms were positioned geometrically (O—H = 0.82, C—H = 0.93 Å) and were included in the refinement in the riding model approximation, with *U*_iso_(H) = x*U*_eq_(C, O), where x = 1.2 for all H-atoms.

### Crystal data

**Table:**

C_14_H_9_Cl_2_NO_2_	*Z* = 2
*M**_r_* = 294.12	*F*(000) = 300
Triclinic, *P*1	*D*_x_ = 1.528 Mg m^−^^3^
Hall symbol: -P 1	Mo *K*α radiation, λ = 0.71073 Å
*a* = 7.4065 (2) Å	Cell parameters from 2048 reflections
*b* = 7.6176 (3) Å	θ = 2.7–25.2°
*c* = 11.5330 (4) Å	µ = 0.50 mm^−^^1^
α = 86.946 (2)°	*T* = 296 K
β = 80.433 (1)°	Prism, light yellow
γ = 85.833 (2)°	0.32 × 0.24 × 0.20 mm
*V* = 639.38 (4) Å^3^	

### Data collection

**Table:**

Bruker Kappa APEXII CCD diffractometer	2293 independent reflections
Radiation source: fine-focus sealed tube	2048 reflections with *I* > 2σ(*I*)
graphite	*R*_int_ = 0.023
Detector resolution: 8.10 pixels mm^-1^	θ_max_ = 25.2°, θ_min_ = 2.7°
ω scans	*h* = −8→8
Absorption correction: multi-scan (*SADABS*; Bruker, 2005)	*k* = −9→9
*T*_min_ = 0.903, *T*_max_ = 0.932	*l* = −13→13
9596 measured reflections	

### Refinement

**Table:**

Refinement on *F*^2^	Primary atom site location: structure-invariant direct methods
Least-squares matrix: full	Secondary atom site location: difference Fourier map
*R*[*F*^2^ > 2σ(*F*^2^)] = 0.031	Hydrogen site location: inferred from neighbouring sites
*wR*(*F*^2^) = 0.084	H-atom parameters constrained
*S* = 1.05	*w* = 1/[σ^2^(*F*_o_^2^) + (0.0382*P*)^2^ + 0.1996*P*] where *P* = (*F*_o_^2^ + 2*F*_c_^2^)/3
2293 reflections	(Δ/σ)_max_ = 0.001
175 parameters	Δρ_max_ = 0.22 e Å^−^^3^
0 restraints	Δρ_min_ = −0.31 e Å^−^^3^

### Special details

**Table:**

Geometry. Bond distances, angles *etc*. have been calculated using the rounded fractional coordinates. All su's are estimated from the variances of the (full) variance-covariance matrix. The cell e.s.d.'s are taken into account in the estimation of distances, angles and torsion angles
Refinement. Refinement of *F*^2^ against ALL reflections. The weighted *R*-factor *wR* and goodness of fit *S* are based on *F*^2^, conventional *R*-factors *R* are based on *F*, with *F* set to zero for negative *F*^2^. The threshold expression of *F*^2^ > σ(*F*^2^) is used only for calculating *R*-factors(gt) *etc*. and is not relevant to the choice of reflections for refinement. *R*-factors based on *F*^2^ are statistically about twice as large as those based on *F*, and *R*- factors based on ALL data will be even larger.

### Fractional atomic coordinates and isotropic or equivalent isotropic displacement parameters (Å^2^)

**Table:**

	*x*	*y*	*z*	*U*_iso_*/*U*_eq_	Occ. (<1)
Cl1	0.14614 (7)	0.31116 (6)	0.46678 (5)	0.0613 (2)	
Cl2	0.34568 (7)	−0.30520 (8)	0.66465 (4)	0.0699 (2)	
O1	−0.31523 (16)	0.47023 (16)	−0.11702 (10)	0.0495 (4)	
O2	−0.35921 (16)	0.36164 (19)	0.06779 (10)	0.0561 (4)	
N1	0.24397 (17)	0.05365 (17)	0.13189 (12)	0.0410 (4)	
C1	−0.2595 (2)	0.3841 (2)	−0.03090 (13)	0.0380 (5)	
C2	−0.0681 (2)	0.30632 (19)	−0.04703 (13)	0.0366 (5)	
C3	0.0469 (2)	0.3230 (2)	−0.15456 (14)	0.0428 (5)	
C4	0.2252 (2)	0.2494 (2)	−0.16592 (15)	0.0473 (5)	
C5	0.2899 (2)	0.1604 (2)	−0.07207 (15)	0.0447 (5)	
C6	0.1764 (2)	0.14779 (19)	0.03695 (14)	0.0378 (5)	
C7	−0.0034 (2)	0.21942 (19)	0.04811 (13)	0.0376 (5)	
C8	0.2038 (2)	0.1171 (2)	0.23329 (14)	0.0409 (5)	
C9	0.24798 (19)	0.0188 (2)	0.33878 (14)	0.0377 (5)	
C10	0.2208 (2)	0.0909 (2)	0.44960 (14)	0.0407 (5)	
C11	0.2511 (2)	−0.0074 (2)	0.54930 (14)	0.0462 (6)	
C12	0.3130 (2)	−0.1807 (2)	0.53859 (14)	0.0457 (5)	
C13	0.3461 (2)	−0.2580 (2)	0.43038 (15)	0.0454 (5)	
C14	0.3120 (2)	−0.1581 (2)	0.33285 (14)	0.0413 (5)	
H1	−0.42024	0.51169	−0.09627	0.0594*	0.500
H2	−0.45884	0.41608	0.06763	0.0674*	0.500
H3	0.00466	0.38291	−0.21822	0.0514*	
H4	0.30252	0.26012	−0.23786	0.0568*	
H5	0.40898	0.10905	−0.08158	0.0536*	
H7	−0.08095	0.20897	0.11998	0.0450*	
H8	0.14485	0.22894	0.24079	0.0491*	
H11	0.23000	0.04301	0.62242	0.0555*	
H13	0.39052	−0.37508	0.42399	0.0545*	
H14	0.33213	−0.21006	0.26025	0.0496*	

### Atomic displacement parameters (Å^2^)

**Table:**

	*U*^11^	*U*^22^	*U*^33^	*U*^12^	*U*^13^	*U*^23^
Cl1	0.0635 (3)	0.0463 (3)	0.0721 (3)	0.0077 (2)	−0.0049 (2)	−0.0192 (2)
Cl2	0.0718 (3)	0.0863 (4)	0.0455 (3)	0.0067 (3)	−0.0032 (2)	0.0180 (2)
O1	0.0445 (7)	0.0579 (7)	0.0441 (7)	0.0117 (5)	−0.0093 (5)	0.0021 (5)
O2	0.0403 (7)	0.0810 (9)	0.0423 (7)	0.0139 (6)	−0.0025 (5)	0.0040 (6)
N1	0.0351 (7)	0.0435 (8)	0.0434 (8)	0.0036 (5)	−0.0063 (5)	−0.0009 (6)
C1	0.0381 (8)	0.0398 (8)	0.0363 (8)	0.0016 (6)	−0.0077 (6)	−0.0052 (6)
C2	0.0359 (8)	0.0355 (8)	0.0392 (8)	−0.0010 (6)	−0.0073 (6)	−0.0061 (6)
C3	0.0436 (9)	0.0471 (9)	0.0375 (9)	−0.0008 (7)	−0.0067 (7)	−0.0014 (7)
C4	0.0417 (9)	0.0584 (10)	0.0388 (9)	0.0000 (7)	0.0017 (7)	−0.0041 (8)
C5	0.0354 (8)	0.0494 (10)	0.0474 (10)	0.0042 (7)	−0.0027 (7)	−0.0062 (7)
C6	0.0372 (8)	0.0345 (8)	0.0417 (9)	0.0007 (6)	−0.0070 (6)	−0.0033 (6)
C7	0.0357 (8)	0.0385 (8)	0.0373 (8)	−0.0003 (6)	−0.0028 (6)	−0.0038 (6)
C8	0.0363 (8)	0.0374 (8)	0.0480 (10)	0.0025 (6)	−0.0059 (7)	−0.0019 (7)
C9	0.0302 (7)	0.0396 (8)	0.0420 (9)	−0.0012 (6)	−0.0022 (6)	−0.0033 (7)
C10	0.0317 (8)	0.0407 (9)	0.0484 (9)	−0.0013 (6)	−0.0010 (6)	−0.0087 (7)
C11	0.0404 (9)	0.0592 (11)	0.0379 (9)	−0.0044 (8)	−0.0003 (7)	−0.0088 (8)
C12	0.0379 (8)	0.0575 (10)	0.0393 (9)	−0.0034 (7)	−0.0018 (7)	0.0048 (7)
C13	0.0444 (9)	0.0416 (9)	0.0470 (9)	0.0018 (7)	−0.0010 (7)	0.0012 (7)
C14	0.0416 (8)	0.0426 (9)	0.0380 (8)	0.0009 (7)	−0.0016 (7)	−0.0064 (7)

### Geometric parameters (Å, °)

**Table:**

Cl1—C10	1.7391 (16)	C8—C9	1.466 (2)
Cl2—C12	1.7345 (16)	C9—C14	1.397 (2)
O1—C1	1.2708 (19)	C9—C10	1.396 (2)
O2—C1	1.2603 (19)	C10—C11	1.379 (2)
O1—H1	0.8200	C11—C12	1.371 (2)
O2—H2	0.8200	C12—C13	1.386 (2)
N1—C6	1.418 (2)	C13—C14	1.372 (2)
N1—C8	1.270 (2)	C3—H3	0.9300
C1—C2	1.482 (2)	C4—H4	0.9300
C2—C7	1.387 (2)	C5—H5	0.9300
C2—C3	1.388 (2)	C7—H7	0.9300
C3—C4	1.385 (2)	C8—H8	0.9300
C4—C5	1.381 (2)	C11—H11	0.9300
C5—C6	1.394 (2)	C13—H13	0.9300
C6—C7	1.390 (2)	C14—H14	0.9300
			
Cl1···C12^i^	3.6226 (16)	C12···C10^ix^	3.594 (2)
Cl1···Cl1^ii^	3.5113 (7)	C12···C11^ix^	3.596 (2)
Cl2···O2^i^	3.1100 (12)	C12···Cl1^i^	3.6226 (16)
Cl1···H8	2.7100	C1···H2^v^	2.5700
Cl1···H13^iii^	3.0700	C1···H1^v^	2.6600
Cl2···H7^i^	2.9900	C4···H11^x^	2.9700
Cl2···H13^iv^	3.1200	C7···H8	2.6400
O1···O2^v^	2.6364 (17)	C8···H7	2.6900
O1···C6^vi^	3.3784 (19)	C14···H4^viii^	2.9500
O2···C1^v^	3.380 (2)	H1···H2	1.9700
O2···O1^v^	2.6364 (17)	H1···O1^v^	2.8800
O2···Cl2^i^	3.1100 (12)	H1···O2^v^	1.8300
O1···H2^v^	1.8400	H1···C1^v^	2.6600
O1···H3	2.5200	H2···H1	1.9700
O1···H1^v^	2.8800	H2···O1^v^	1.8400
O1···H8^vi^	2.8900	H2···O2^v^	2.6800
O1···H14^vii^	2.6700	H2···C1^v^	2.5700
O2···H7	2.4400	H3···O1	2.5200
O2···H1^v^	1.8300	H4···H11^x^	2.5100
O2···H2^v^	2.6800	H4···C14^viii^	2.9500
N1···C2^vii^	3.377 (2)	H5···N1^viii^	2.7600
N1···H14	2.5400	H7···O2	2.4400
N1···H5^viii^	2.7600	H7···C8	2.6900
C1···O2^v^	3.380 (2)	H7···H8	2.3700
C2···C2^vi^	3.470 (2)	H7···Cl2^i^	2.9900
C2···C6^vii^	3.599 (2)	H8···Cl1	2.7100
C2···N1^vii^	3.377 (2)	H8···C7	2.6400
C6···C2^vii^	3.599 (2)	H8···H7	2.3700
C6···C7^vii^	3.415 (2)	H8···O1^vi^	2.8900
C6···O1^vi^	3.3784 (19)	H11···C4^xi^	2.9700
C7···C7^vii^	3.572 (2)	H11···H4^xi^	2.5100
C7···C6^vii^	3.415 (2)	H13···Cl1^xii^	3.0700
C10···C12^ix^	3.594 (2)	H13···Cl2^iv^	3.1200
C10···C11^i^	3.596 (2)	H14···N1	2.5400
C11···C12^ix^	3.596 (2)	H14···O1^vii^	2.6700
C11···C10^i^	3.596 (2)		
			
C1—O1—H1	109.00	C10—C11—C12	118.77 (15)
C1—O2—H2	109.00	Cl2—C12—C13	119.88 (12)
C6—N1—C8	117.97 (13)	Cl2—C12—C11	118.61 (12)
O1—C1—O2	123.13 (14)	C11—C12—C13	121.50 (15)
O1—C1—C2	118.55 (13)	C12—C13—C14	118.57 (14)
O2—C1—C2	118.32 (14)	C9—C14—C13	122.33 (15)
C1—C2—C3	120.96 (13)	C2—C3—H3	120.00
C3—C2—C7	120.10 (14)	C4—C3—H3	120.00
C1—C2—C7	118.93 (13)	C3—C4—H4	119.00
C2—C3—C4	119.23 (15)	C5—C4—H4	120.00
C3—C4—C5	121.03 (15)	C4—C5—H5	120.00
C4—C5—C6	119.87 (14)	C6—C5—H5	120.00
N1—C6—C7	121.59 (14)	C2—C7—H7	120.00
N1—C6—C5	119.16 (13)	C6—C7—H7	120.00
C5—C6—C7	119.16 (14)	N1—C8—H8	119.00
C2—C7—C6	120.56 (14)	C9—C8—H8	119.00
N1—C8—C9	121.74 (14)	C10—C11—H11	121.00
C8—C9—C14	120.23 (14)	C12—C11—H11	121.00
C8—C9—C10	123.03 (14)	C12—C13—H13	121.00
C10—C9—C14	116.66 (14)	C14—C13—H13	121.00
Cl1—C10—C11	117.26 (12)	C9—C14—H14	119.00
Cl1—C10—C9	120.59 (12)	C13—C14—H14	119.00
C9—C10—C11	122.16 (14)		
			
C8—N1—C6—C5	139.76 (15)	C5—C6—C7—C2	−1.6 (2)
C8—N1—C6—C7	−43.9 (2)	N1—C8—C9—C10	174.01 (15)
C6—N1—C8—C9	172.33 (13)	N1—C8—C9—C14	−9.3 (2)
O1—C1—C2—C3	−1.6 (2)	C8—C9—C10—Cl1	−4.6 (2)
O1—C1—C2—C7	176.92 (14)	C8—C9—C10—C11	175.35 (14)
O2—C1—C2—C3	178.44 (15)	C14—C9—C10—Cl1	178.61 (11)
O2—C1—C2—C7	−3.0 (2)	C14—C9—C10—C11	−1.5 (2)
C1—C2—C3—C4	179.60 (14)	C8—C9—C14—C13	−176.55 (14)
C7—C2—C3—C4	1.1 (2)	C10—C9—C14—C13	0.4 (2)
C1—C2—C7—C6	−178.74 (14)	Cl1—C10—C11—C12	−178.91 (12)
C3—C2—C7—C6	−0.2 (2)	C9—C10—C11—C12	1.2 (2)
C2—C3—C4—C5	−0.1 (2)	C10—C11—C12—Cl2	−178.48 (12)
C3—C4—C5—C6	−1.7 (2)	C10—C11—C12—C13	0.3 (2)
C4—C5—C6—N1	179.03 (14)	Cl2—C12—C13—C14	177.42 (12)
C4—C5—C6—C7	2.6 (2)	C11—C12—C13—C14	−1.3 (2)
N1—C6—C7—C2	−178.01 (14)	C12—C13—C14—C9	1.0 (2)

Symmetry codes: (i) −*x*, −*y*, −*z*+1; (ii) −*x*, −*y*+1, −*z*+1; (iii) *x*, *y*+1, *z*; (iv) −*x*+1, −*y*−1, −*z*+1; (v) −*x*−1, −*y*+1, −*z*; (vi) −*x*, −*y*+1, −*z*; (vii) −*x*, −*y*, −*z*; (viii) −*x*+1, −*y*, −*z*; (ix) −*x*+1, −*y*, −*z*+1; (x) *x*, *y*, *z*−1; (xi) *x*, *y*, *z*+1; (xii) *x*, *y*−1, *z*.

### Hydrogen-bond geometry (Å, °)

**Table:**

*D*—H···*A*	*D*—H	H···*A*	*D*···*A*	*D*—H···*A*
O1—H1···O2^v^	0.82	1.83	2.6364 (17)	170
O2—H2···O1^v^	0.82	1.84	2.6364 (17)	162
C8—H8···Cl1	0.93	2.71	3.0934 (17)	105

Symmetry codes: (v) −*x*−1, −*y*+1, −*z*.
